# Substitute for the Elastic Stocking

**Published:** 1875-12

**Authors:** Walter H. O’Neal

**Affiliations:** Physician to Adams County Almshouse, Gettysburg, Pa.


### SUBSTITUTE FOR THE ELASTIC STOCKING.

BY WALTER H. O'NEAL,

*Physician to Adams County Almshouse, Gettysburg, Pa.*

The following device was originated by my father, Dr. J. W. C. O'Neal, and has been used in the wards of the hospital under my care. It is cheap, efficient, and easily prepared:

To a limb requiring support, a well-fitting bandage is applied, over which and on either side a coat of well-made and strained starch is added. Then pasteboard softened in liquid starch is applied, leaving a line of unstiffened material, front and back. Over this is added a bandage, which, in turn, is secured by paste. The limb is now suffered to lie quiet until the apparatus hardens.

To remove the hardened bandage, cut along the unstiffened seams, and dress them bookbinder fashion with strips of pasted muslin. Cover the inside with pasted strips to prevent creasing. In this way, and in two parts, a perfect case is made for a diseased limb, which may be removed and reapplied with little trouble, and by almost anybody, as often as necessary. The case is kept free from impurities, smell, etc., by sponging on the inside with a solution of carbolic acid (ʒ j to a quart of water). It is kept clean by first applying an ordinary bandage to the limb, and securing it by the ordinary roller.

This appliance is not designed to take the place of the elastic stocking among a class of persons who can afford the same outlay, but as a substitute for it, for use by the country practioner, and in the wards of a hospital, away from the ordinary conveniences to which city professional gentlemen have access.—*N. Y. Med. Jour.*

